# Report of the Salaries Committee of the College of Nursing

**Published:** 1919-06-07

**Authors:** 


### SALARIES AND CONDITIONS OF EMPLOYMENT OF NURSES.

### REPORT OF THE SALARIES COMMITTEE OF THE COLLEGE OF NURSING.

This Report constitutes probably the most important official document on the subjects dealt with which has yet been issued through the Press. It is a very voluminous document, and if printed in its entirety, in small type, would occupy from seven to eight pages of The Hospital. One Print by some means obtained access to this document before it was circulated to the Press, which led to the publication of a very imperfect statement, calculated to confuse rather than instruct the nursing profession. The College of Nursing may suffer serious injury, both in credit and reputation, if the leakages which have taken place of late in regard to its business and other matters are not promptly and effectually prevented for the future. The Report itself is full of original and complete information of the highest value. Its contents should be taken to heart by the managers and controllers of all institutions, both public and private, which employ or are in any way responsible for nursing or nurses throughout the British Islands. Arrangements should be immediately made by the College to circulate this Report and make it readily obtainable by all working nurses, and everybody interested in their welfare, progress, education, and protection.

The terms of reference to the Salaries and Employment Committee were: "To consider and report upon the Conditions of Employment and upon the Salaries of Nurses throughout the United Kingdom (Mental Nurses excepted) both during and after training, and whether engaged in institutional, district, or any other form of nursing."

We have held over the preliminary statements, with the detailed figures of two questionnaires, but give elsewhere a summary of all the figures they contain. (See "Round the Hospitals," page 247.) We are further omitting a whole page of illustrations (*i.e.* descriptions), which demonstrate that an average duty week of forty-eight hours is both possible and practicable. These we hope to give in our next issue, with tables showing scales of salaries, nurses' letters on off-duty, accommodation, and on pensions, with the committee's remarks on superannuation.

The substance of the report, which we now publish, will demonstrate to the whole nursing profession how thoroughly well the College authorities have discharged their responsible duties in appointing and selecting the members of this able committee. The capacity and grip of which the report affords evidence must greatly extend the reputation and influence of the College, providing its Council takes this Report in hand at once and agrees to provide the necessary lead and takes energetic action without which, despite its thoroughness, the Report might easily become a dead letter. The College of Nursing has now the high privilege of devising the means to enforce the reforms, and to materialise the many excellent suggestions for improvement in the conditions of service, to be found in the Report. The sooner these reforms and improvements are enforced throughout the nursing world the sooner will the position of trained nurses, everywhere, be improved. The outlook for each of them should then be made what the nation hopes and intends it to become.

In a subsequent issue we hope to take up in detail many points arising out of the Report. Our privilege and duty to-day is to congratulate Mrs. Field and the members of her committee upon the invaluable work they have accomplished, with an expedition and thoroughness which does every member of it the highest credit. This committee has earned, and should receive, the fullest and most grateful acknowledgments of the whole nursing profession, together with everybody interested in its advancement and reform.

### Conditions of Employment,

Hours.—The highest number of hours of duty shown on the returns is 71 hours a week on day duty and 84 hours a week on night duty. The lowest is 52½ hours a week day duty and 59¼ hours night duty. One nurse writes that her duty hours are 86½ a week, but from the time-table enclosed her average weekly hours taken over a period of two months are 78. Another nurse states her hours on duty to be 86 a week and on night duty 84 a week.

It is difficult to calculate from the time-tables sent the average weekly hours of duty of the nurses. Many hospitals give simply the hours off duty. Some portion of this time may, however, be taken up with lectures, writing of reports, or presiding at meals. Three Poor-Law institutions and one fever hospital give the actual number of weekly hours on duty. In all other cases the off-duty hours are given.

The Committee suggests that time-tables be framed to show the actual hours on duty, and that nurses be advised *at least one week* ahead when the periods of off-duty will fall, so that they may be able to make arrangements for off-duty days.

It is the custom in most hospitals that the sisters work less hours than the staff nurses, and in some that the staff nurses work less hours than the probationers. Night duty is invariably longer than day duty. In two hospitals only is it expressly stated that the night nurses are compelled to take a meal in the dining-room between 12 and 3 a.m., when a maid attends to their wants, and when they must take at least one hour off-duty.

The Committee recommends that an average week of 48 hours of duty be fixed as a maximum for all nurses—such average to be taken over a period of not more than three months.

Included in the average duty week of 48 hours are: (*a*) All meals taken in the wards. (*b*) All lectures which are obligatory. (*c*) All correcting of theoretical work of probationers. (*d*) All teaching or lecturing which is obligatory. (*e*) The writing of all reports. (*f*) The meal-times of those whose duty it is to preside at meals. (*g*) The keeping of all records. (*h*) All work done in private rooms which is obligatory, such as making of beds and dusting. It does not include time taken to get to and from the wards to the dining-room or Nurses' Home.

As the time-tables in the various institutions differ considerably, it is felt that there need be little difficulty in readjusting the hours of duty to give the average of 48 hours weekly suggested above.

From the evidence before the Committee it would appear that lecture-hours are not always arranged at suitable times in several hospitals. One assistant matron gives all her lectures at 7 p.m., when she has been on duty 12½ hours. This is equally injurious to the teacher and the taught.

### Private Nursing Homes.

The same number of hours weekly is recommended for private nursing homes as for institutions.

**Salaries and Conditions of Employment—(*cont.*)**

Private Nursing Staffs, Co-operations.

It is difficult to fix the hours of a nurse doing private nursing, but nursing co-operations are recommended to advise clients that, unless the case needs constant attention, the average week should be 48 hours.

Queen's Nurses, District Nurses.

It is strongly felt that the 48-hours week laid down by the Queen Victoria's Jubilee Institute for Nurses should be accepted by all Associations employing district nurses, and that the necessary steps should be taken to see that this rule is adhered to by all affiliated societies.

School Nurses, Welfare Workers.

The hours fixed by the Corporations and County Councils are, generally speaking, 42 a week.

Meal Times.

If the following meals fall within the periods of on duty of the nurses, the following minimum time should be allowed:—

(*a*) Breakfast, half-hour.

(*b*) Midday meal one hour, and evening meal half-hour; *or* midday meal half-hour, and evening meal one hour.

(*c*) Tea, ½ hour.

### Staff.

The Committee considers that in hospitals with an average of 300 beds or more than 300 beds occupied, the Matron should have a staff of not less than—(1) Assistant matron; (2) a teaching sister; (3) a home sister; (4) a housekeeper; (5) a night superintendent and assistant; (6) a secretarial assistant; (7) a sufficient number of sisters to allow not less than one sister to every 30 beds; (8) a day staff nurse and a night staff nurse to each ward of 30 beds; (9) not less than four probationers to each ward of 30 beds.

In hospitals with 100 to 300 beds occupied probably a smaller administrative staff would be sufficient.

In hospitals with 50 to 100 beds occupied the matron's staff should be not less than—(1) An assistant matron or home sister; (2) a sufficient number of sisters to allow one sister to 30 beds; (3) two staff nurses to 30 beds.

The matrons of hospitals of less than 50 beds occupied deserve special consideration. It is recommended that their staff be not less than—(1) A sister; (2) two staff nurses.

The Salaries Committee fully recognises the difficulties which will confront the smaller hospitals, when they cannot undertake the training of probationers, and considers that they can best be overcome by the scheme of affiliation before suggested.

Night Nurses.

No nurse should be allowed to be on duty for a period of longer than three months.

Staff Nurses.

Staff nurses should possess a certificate of three years' training at a recognised training-school, and should have had further experience before being appointed on to the staff of the hospital.

Charge Nurses.

These nurses should possess a certificate of three years' training at a recognised training-school. It is recommended that training-schools should provide facilities for charge nurses to gain good experience and to take further certificates.

Probationers.

The general hospitals take probationers at the age of 23, and in some cases at 21, so that an interval of several years passes between school life and probation. With this period the hospitals do not concern themselves. Evidence before the Committee shows that girls have entered on other careers and become well advanced before beginning as probationers in a general hospital. Women of 23 hold responsible positions in other professions and command salaries up to £250 a year. Women doctors are often qualified at the age of 23. It is recommended that the minimum age at which probationers are admitted for training into general hospitals be 21. As the number of professions open to women increases daily, the difficulty of obtaining probationers will become greater unless the remuneration after training is increased and the general conditions of service improved.

Agreements.

The agreement signed by the probationer should ensure to her adequate training and experience in the medical and surgical nursing of men, women, and children.

Trained nurses should be warned against undertaking any work under agreement with any association, society, or private nursing home, unless such agreement has been inquired into by a competent authority.

Holidays.

A period *of not less than* one month in the year should be allowed all members of the nursing profession.

Rules of Hospitals and Nursing Institutions.

All rules and regulations sent out by nursing institutions to prospective probationers should be brought up to date and made as simple and clear as possible.

School Nurses.

From evidence before the Committee it is strongly felt that the work of the school nurse does not receive sufficient recognition. In certain districts she is asked to undertake the duties of an attendance officer, which is an arrangement to be strongly deprecated.

Working Arrangements and Equipment of Hospitals.

Nurses state that they have often to confront the difficulty of obtaining adequate hot-water supplies, and that there is endless waste of time and strength in carrying water from the gas jets by the bucketful because the main supply runs cold. In one instance recorded a nurse had to carry as many as ten buckets of water every night up a long flight of steps in order to supplement the bath supply.

The appointment of male "hospital orderlies" is strongly recommended in the older hospitals, where the engineer is unable to overcome difficulties due to the faulty construction of the building.

Health of Nurses.

It is felt that definite steps should be taken to ensure nurses not going on duty when unfit to do so. They should be encouraged to report this themselves to the Matron, who will make the proper arrangements. If the staff of the institution is not large enough the Matron should have the power to take such steps as she considers necessary to augment her staff.

### Salaries.

The scale of salaries is based on an allowance being made for—

(*a*) Laundry.

(*b*) Indoor uniform.

(*c*) Travelling expenses in the case of isolation hospitals more than three miles from the centre of the town or railway station.

Laundry.

When full laundry is not allowed, a minimum of 3s. 6d. per week should be given or the equivalent amount of laundry allowance based on outside prices.

Uniform.

An allowance of not less than the material for four dresses, four caps, twelve aprons, twelve collars, and twelve pairs of cuffs should be made the first year, and half the allowance in subsequent years.

If in the first three months of probation the probationer supplies her own uniform to the pattern of the hospital uniform, then if she enter into agreement with the hospital, the equivalent sum should be refunded to her.

When an allowance is made in money for uniform, a sum of not less than £8 should be given the first year, and £4 in subsequent years.

### Salaries and Conditions of Employment—(*cont.*)

TRAVELLING EXPENSES.

In the case of hospitals more than three miles from the nearest railway station, when arrangements are not made by the hospital for conveyance, an extra allowance of not less than £3 a year should be made to each of the staff to cover the necessary expenditure involved.

SCHOOL NURSES, WELFARE WORKERS, DISTRICT NURSES.

All travelling expenses incurred in the execution of work should be refunded. When a bicycle is not provided, a minimum allowance of 1½d. a mile should be made for wear and tear of the machine.

CONVALESCENT HOMES.

The average salary of trained nurses in these institutions, of which eight were considered, is £44. It is felt that, whilst nurses undertaking this work should be fully trained, it might be undertaken by those older or less robust.

HEALTH VISITORS, WELFARE WORKERS UNDER COUNCILS.

The salaries vary from £80 to £178, not including war bonus. The minimum salary should be £120 with uniform, or £130 without uniform.

Allowances should be made for travelling necessary in the execution of work, and when a nurse is compelled to be away overnight a further special allowance should be made.

NURSING ASSOCIATIONS.

Of these, sixty-five were considered. There is a wide difference existing in salaries and in conditions of employment under the various nursing Associations. Salaries of trained nurses range from £30 to £60 resident, and from £95 to £125 non-resident. District nursing is an important branch of the nursing profession. The district nurse is called upon at all times and in all weathers. It is felt that nurses who have completed their training should begin with a minimum of £50 resident (uniform and laundry provided) or £120 non-resident, with uniform and laundry provided.

When the salary is fixed at a resident figure with allowances for board and lodgings, the minimum allowance should be £70 a year or £45 for board and the rent of two furnished rooms with fire, light, and attendance.

NAVAL AND MILITARY NURSES.

No nurse with a certificate of three years' general training should be paid less than £50 yearly, with an allowance as set out above for uniform and laundry.

When nurses are on active service an allowance equal to not less than that made to a junior officer should be given in addition.

NURSES IN WORKS.

Ten works were considered. The average salary paid is £147, without any allowances. The salaries range from £104 (not including war bonus), with uniform and laundry allowance to £200, and meals when on duty. The highest number of hours worked is 56—the average 45½ per week.

It is considered that the minimum salary should be £120 with uniform and laundry, or £140 without laundry, uniform or meals, or £60 resident.

The holidays vary from seven days, with public holidays, to fourteen days and public holidays.

PRIVATE NURSES.

Nurses working privately should not charge less than two guineas a week, with an allowance of 3*s.* 6*d.* for laundry and the usual travelling expenses.

Nurses working on the private staff of institutions or on Associations should be able to clear at least £60 a year, if resident, and the same amount, after expenses for rooms and board between cases have been deducted, if non-resident.

When nurses work on a co-operative system the percentage on fees should be not more than 7½ per cent.

If the nurse doing private nursing leaves for any cause, other than by her own desire, within any given week, then the part of a week should be paid for as one whole week.

It is strongly urged that in connection with district or private nursing homes and co-operations a certain number of visiting nurses should be employed to do daily visiting at a charge to each patient per week or per visit.

SANATORIA.

Six returns were considered. The salaries range from £40 to £45. It is suggested that, where possible, the sanatoria should be worked from a large consumptive hospital. The nurses would thus be interchangeable and the overhead charges greatly reduced.

SCHOOL NURSES UNDER COUNCILS.

The payment of school nurses ranges from £75 to £120 non-resident. The trained nurse should command the same minimum salary as the trained teacher under the same Council. Uniform should be optional; but, if provided, a reduction of not more than £8 yearly should be made.

Travelling expenses should be paid, and, if a bicycle is not provided, an allowance of not less than 1½d. a mile should be made or tram tickets given.

### Conclusion.

In conclusion, the Salaries Committee would appeal to all in the nursing profession not to aim at "standardisation" more than is necessary to uphold the status of the profession.

The Committee is of opinion that there is on the part of the hospitals, infirmaries, and public nursing institutions, and the medical officers of health, a keen sympathy with all movements tending to improve the status of the nurse.

The Committee strongly feels that the hospitals and Poor-Law infirmaries are the training-schools of the nurses, and any improvements and additions in the way of teaching should come from them rather than from any outside schools or universities.

It is recommended that, as the children's hospitals and hospitals for infectious diseases demand a three years' general training for the higher posts on their staffs, these hospitals should be in affiliation with as many general hospitals as practicable, so that probationers going to them should not be entering upon a "blind alley occupation."

Similarly in the case of the hospitals for special diseases, it is recommended that nurses with a three years' general training only shall be employed, for whom courses of instruction in the special branches should be established.

(Signed)

LOUISE F. FIELD (*Chairman*). E. S. HALDANE.
SYBIL DE S. BRASSEY. HENRIETTA CORNER.
MAY WHITTY. A. C. GIBSON.
JANET E. COURTNEY. ROSA M. BARRETT.
KATIE E. S. MACDONALD. LOUIS H. M. DICK.

